# Association Between Vitamin D Deficiency and Glycemic, Lipid, and Adiposity Markers in Older Adults: A Nationally Representative Study

**DOI:** 10.3390/metabo16040270

**Published:** 2026-04-16

**Authors:** Yong-Joon Kim, Kyeongmin Jang

**Affiliations:** 1 Department of Emergency Medicine, Seoul Metropolitan Government Boramae Medical Center, Seoul National University, Seoul 07061, Republic of Korea; brmeremt130@naver.com; 2 Department of Nursing, College of Health Sciences, Daejin University, Pocheon-si 11159, Republic of Korea

**Keywords:** vitamin D deficiency, insulin resistance, lipid metabolism, central obesity, older adults, cardiometabolic risk, population-based study

## Abstract

Background/Objectives: Vitamin D plays an important role in glucose metabolism, lipid regulation, and inflammatory processes, and has been implicated in cardiometabolic health. However, its associations with specific metabolic biomarkers remain inconsistent, particularly in older adults. This study aimed to examine whether vitamin D deficiency is differentially associated with multiple metabolic biomarkers in a nationally representative sample of older adults. Methods: This cross-sectional study used data from the 2024 Korea National Health and Nutrition Examination Survey, including 1806 adults aged ≥65 years. Vitamin D deficiency was defined as serum 25-hydroxyvitamin D levels < 20 ng/mL. Metabolic biomarkers included fasting glucose, glycated hemoglobin (HbA1c), triglycerides, C-reactive protein (CRP), high-density lipoprotein cholesterol (HDL-C), waist circumference, and body mass index (BMI). Complex sample linear regression analyses were performed with sequential adjustment for sociodemographic factors, health behaviors, and comorbidities. Results: In unadjusted analyses, vitamin D deficiency was associated with adverse metabolic profiles, including higher fasting glucose, HbA1c, triglycerides, waist circumference, and CRP levels, and lower HDL-C levels. After adjustment for sociodemographic factors, health behaviors, and comorbidities, significant associations remained for HbA1c (β = 0.10, *p* = 0.034), triglycerides (β = 0.10, *p* = 0.003), and waist circumference (β = 1.21, *p* = 0.040). No significant associations were observed for fasting glucose, HDL-C, CRP, or BMI. Conclusions: Vitamin D deficiency was independently associated with poorer long-term glycemic status, hypertriglyceridemia, and central adiposity in older adults, but not with other metabolic markers after adjustment. These findings suggest that the metabolic correlates of vitamin D deficiency may be domain-specific rather than generalized. Longitudinal and interventional studies are needed to clarify causality and underlying mechanisms.

## 1. Introduction

Cardiometabolic disorders, including diabetes, dyslipidemia, and central obesity, are major contributors to morbidity and mortality in older adults [1,2]. With rapid population aging, the burden of these conditions has increased substantially, particularly among community-dwelling older individuals [2,3]. These metabolic abnormalities frequently coexist and share common pathophysiological mechanisms, including insulin resistance, chronic low-grade inflammation, and dysregulated lipid metabolism, which collectively increase the risk of cardiovascular disease and mortality [4,5].

Vitamin D, traditionally recognized for its role in calcium homeostasis and bone metabolism, has increasingly been identified as a pleiotropic regulator of metabolic health [6]. Vitamin D receptors are widely distributed in pancreatic β-cells, adipose tissue, and skeletal muscle, suggesting a broader role in glucose and lipid metabolism. Experimental and clinical evidence indicates that vitamin D enhances insulin secretion, improves insulin sensitivity, and modulates inflammatory pathways, thereby influencing cardiometabolic homeostasis [6]. In addition, vitamin D has been shown to regulate adipogenesis and adipocyte function, linking it to obesity-related metabolic disturbances [7]. Accordingly, vitamin D deficiency has been associated with multiple metabolic abnormalities implicated in cardiometabolic disease [6].

A growing body of epidemiological research has explored the association between vitamin D status and cardiometabolic risk factors. Recent studies suggest that lower circulating 25-hydroxyvitamin D levels are associated with an increased risk of type 2 diabetes and adverse cardiometabolic profiles, including obesity and dyslipidemia [8]. Population-based studies have further reported inverse associations between vitamin D levels and metabolic syndrome, particularly with respect to abdominal obesity and elevated triglycerides [9]. In Korean adults, recent evidence has also shown that vitamin D status is related to cardiometabolic risk clustering and inflammatory burden, further supporting its potential role in metabolic health [10]. In addition, observational findings indicate that vitamin D deficiency is associated with insulin resistance, a central feature of metabolic dysregulation [11].

Despite these findings, the relationship between vitamin D and cardiometabolic health remains inconsistent. Some interventional and meta-analytic studies have reported modest improvements in glycemic control and lipid profiles following vitamin D supplementation, including reductions in HbA1c and triglyceride levels [12]. However, other studies have found limited or no significant effects on glycemic outcomes, lipid metabolism, or adiposity after accounting for confounding factors such as obesity, lifestyle, and baseline vitamin D status [13]. These inconsistencies may reflect heterogeneity in study populations, differences in study design, and the complex interplay between vitamin D and metabolic pathways.

Another limitation of the existing literature is that many studies have focused on individual metabolic outcomes rather than evaluating multiple biomarkers simultaneously. Cardiometabolic risk is a multidimensional construct involving glucose regulation, lipid metabolism, inflammation, and adiposity. Therefore, examining these domains together may provide a more comprehensive understanding of the metabolic role of vitamin D. In addition, relatively few studies have used nationally representative datasets with appropriate complex sampling designs, particularly in older populations, limiting the generalizability of findings. Older adults represent a high-risk group with both an increased prevalence of vitamin D deficiency and greater susceptibility to metabolic disorders, yet remain underrepresented in many large-scale analyses.

Therefore, this study aimed to examine the association between vitamin D deficiency and multiple metabolic biomarkers, including glycemic, lipid, inflammatory, and anthropometric indicators, in a nationally representative sample of older adults. Older adults were specifically targeted because they represent a high-risk group with both a higher prevalence of vitamin D deficiency and an increased burden of cardiometabolic disorders. In addition, age-related physiological changes, such as reduced cutaneous synthesis of vitamin D and alterations in body composition, may influence both vitamin D status and metabolic health. By applying complex sample analyses to population-based data, this study sought to provide a comprehensive evaluation of these associations and to clarify the relationship between vitamin D deficiency and key metabolic pathways in older populations.

## 2. Materials and Methods

### 2.1. Study Design and Data Source

This study employed a cross-sectional design using data from the Korea National Health and Nutrition Examination Survey (KNHANES). The dataset used in this study was collected in 2024 and released in December 2025. KNHANES is a nationally representative surveillance system of the non-institutionalized Korean population and has been conducted as an annual survey using a complex, multistage probability sampling design to generate nationally representative health and nutrition statistics in Republic of Korea. More recent overviews have also highlighted the continued expansion and public availability of KNHANES data resources [14]. KNHANES collects data through standardized health interviews, health examinations, and laboratory tests conducted by trained personnel. In the present study, all variables were extracted from the KNHANES dataset. Sociodemographic characteristics and health behavior variables were obtained from self-reported questionnaires, whereas anthropometric measures and metabolic biomarkers were based on direct physical examination and laboratory assessment.

### 2.2. Study Participants

Among the 6997 participants included in the 2024 KNHANES dataset, individuals aged 65 years or older were considered eligible for this study (*n* = 1951). Participants were included if they had available data on serum vitamin D levels and all relevant metabolic biomarkers. Individuals with missing values for key variables, including vitamin D, metabolic biomarkers, or covariates, were excluded from the analysis. After applying these criteria, a total of 1806 participants were included in the final analytic sample. All analyses incorporated sampling weights to account for the complex survey design and to ensure nationally representative estimates.

### 2.3. Vitamin D Status

The main independent variable was vitamin D status. Serum vitamin D concentration was assessed using 25-hydroxyvitamin D [25(OH)D], which is the accepted biomarker for evaluating vitamin D status. Vitamin D deficiency was defined as a serum 25(OH)D level < 20 ng/mL. This threshold remains widely used in recent vitamin D literature, although recent guidelines have noted that the optimal target concentration for disease prevention remains under debate. Participants were categorized into vitamin D-deficient and non-deficient groups accordingly [15].

### 2.4. Metabolic Biomarkers

The dependent variables included fasting glucose (mg/dL), glycated hemoglobin (HbA1c, %), triglycerides (mg/dL), C-reactive protein (CRP, mg/L), high-density lipoprotein cholesterol (HDL-C, mg/dL), waist circumference (cm), and body mass index (BMI, kg/m^2^). Laboratory biomarkers were obtained from blood samples collected and analyzed by KNHANES using standardized procedures, and fasting glucose and triglycerides were based on fasting blood samples. Anthropometric variables, including waist circumference, height, and weight, were measured directly by trained personnel using standardized protocols. BMI was calculated as weight in kilograms divided by height in meters squared. These biomarkers are commonly used indicators of metabolic and cardiometabolic risk in older adults. Because triglycerides and C-reactive protein exhibited skewed distributions, natural logarithmic transformation was applied prior to analysis to improve normality.

### 2.5. Covariates

Covariates included sociodemographic characteristics, health behaviors, and comorbid conditions. Sociodemographic variables included age (years), sex (male/female), residence (urban/rural), household income, and education level. Residence was categorized as urban or rural based on administrative classification. Household income was categorized into quartiles (Q1–Q4) based on the KNHANES household income quartile variable, which is derived from monthly equivalized household income (monthly household income divided by the square root of household size). Education level was categorized as ≤elementary school, middle school, high school, or ≥college.

Health behavior variables included monthly alcohol consumption, current smoking status, and aerobic physical activity. These variables were assessed using self-reported questionnaires. Monthly alcohol consumption was defined as drinking alcohol at least once per month (yes/no), and current smoking status was categorized as yes or no. Aerobic physical activity was defined according to KNHANES guidelines as engaging in at least 150 min of moderate-intensity physical activity or 75 min of vigorous-intensity physical activity per week, or an equivalent combination of both.

Comorbidities included hypertension and diabetes mellitus, which were defined based on physician diagnosis, current treatment, or standardized KNHANES criteria. These variables were selected as potential confounders based on previous studies demonstrating their associations with both vitamin D status and metabolic risk [16,17].

Obesity- and inflammation-related variables, including body mass index, waist circumference, and C-reactive protein, were not included as covariates because they were analyzed as outcome variables in this study. Including these variables as covariates could result in over-adjustment and potentially obscure the associations of interest.

### 2.6. Statistical Analysis

All statistical analyses were performed using IBM SPSS Statistics (version 29.0, IBM Corp., Armonk, NY, USA), applying complex sample design procedures to account for the stratification, clustering, and sampling weights of KNHANES. These procedures were used to generate nationally representative estimates and appropriate standard errors reflecting the multistage probability sampling design. Descriptive statistics were presented as weighted percentages with standard errors (SE) for categorical variables and weighted means with SE for continuous variables. Differences in participant characteristics according to vitamin D status were assessed using the Rao–Scott chi-square test for categorical variables and complex sample general linear models for continuous variables. To examine the association between vitamin D status and metabolic biomarkers, complex sample linear regression analyses were conducted, with vitamin D status included as a dichotomous independent variable (deficient vs. non-deficient) and metabolic biomarkers treated as continuous dependent variables. Model 1 was unadjusted. Model 2 was adjusted for sociodemographic variables (age, sex, residence, household income, and education level). Model 3 was additionally adjusted for health behaviors (smoking, alcohol consumption, and aerobic physical activity) and comorbidities (hypertension and diabetes mellitus). The results were presented as β coefficients, 95% confidence intervals (CI), and *p*-values. Statistical significance was set at *p* < 0.05.

### 2.7. Ethical Considerations

KNHANES was conducted by the Korea Disease Control and Prevention Agency (KDCA) in accordance with the Declaration of Helsinki and was approved by the KDCA Institutional Review Board (IRB No. 2022-11-16-R-03). Written informed consent was obtained from all participants during the original survey. The present study was based on a secondary analysis of publicly available KNHANES data collected in 2024 and released in December 2025. Because the dataset was fully de-identified and contained no personally identifiable information, additional institutional review board approval was not required for this study.

## 3. Results

### 3.1. General Characteristics of Participants

The general characteristics of the study population are presented in Table 1. A total of 1806 older adults were included, with a mean age of 72.78 ± 0.18 years. Women accounted for 54.0% of the sample, and 73.8% resided in urban areas. Socioeconomically, 37.2% were in the lowest household income quartile, and 43.1% had an education level of elementary school or lower. In terms of health behaviors, 64.7% reported no monthly alcohol consumption, 91.9% were non-smokers, and 67.6% did not engage in aerobic physical activity. The prevalence of hypertension and diabetes mellitus was 54.0% and 24.8%, respectively. The mean serum vitamin D level was 29.88 ± 0.38 ng/mL. The mean fasting glucose level was 107.42 ± 0.57 mg/dL, and the mean HbA1c was 5.97 ± 0.02%. The mean HDL-cholesterol level was 56.41 ± 0.41 mg/dL, and the mean waist circumference was 85.69 ± 0.27 cm (Table 1).

### 3.2. Differences in Characteristics According to Vitamin D Status

Among the study population, 409 participants (22.6%) were classified as vitamin D deficient. Differences in participant characteristics according to vitamin D status are presented in Table 2. Compared with participants without vitamin D deficiency, those with deficiency were older and more likely to be male. They also had a higher prevalence of current smoking and diabetes mellitus and were less likely to engage in aerobic physical activity. In terms of metabolic profiles, participants with vitamin D deficiency showed less favorable profiles, including higher fasting glucose, HbA1c, triglycerides, and waist circumference, as well as lower HDL-cholesterol levels. No significant differences were observed for residence, household income, education level, monthly alcohol consumption, hypertension, or body mass index.

### 3.3. Association Between Vitamin D Deficiency and Metabolic Biomarkers

The associations between vitamin D deficiency and metabolic biomarkers are presented in Table 3. In the unadjusted model, vitamin D deficiency was associated with higher levels of fasting glucose, HbA1c, triglycerides, and waist circumference, and with lower HDL-cholesterol levels. After adjustment for sociodemographic factors, the associations remained significant for HbA1c, triglycerides, and waist circumference, whereas those for fasting glucose and HDL-cholesterol were attenuated. In the fully adjusted model, vitamin D deficiency remained significantly associated with higher HbA1c (β = 0.10, 95% CI: 0.01–0.20, *p* = 0.034), higher triglyceride levels (β = 0.10, 95% CI: 0.04–0.17, *p* = 0.003), and greater waist circumference (β = 1.21, 95% CI: 0.06–2.37, *p* = 0.040). No significant associations were observed for fasting glucose, HDL-cholesterol, C-reactive protein, or body mass index. Detailed regression coefficients are presented in Table 3.

## 4. Discussion

This study aimed to examine the association between vitamin D deficiency and multiple metabolic biomarkers in a nationally representative sample of older adults. The main findings showed that vitamin D deficiency remained independently associated with higher HbA1c, triglyceride levels, and waist circumference after full adjustment, whereas the associations with fasting glucose, HDL-cholesterol, C-reactive protein, and body mass index were no longer significant. These findings suggest that the metabolic correlates of vitamin D deficiency in later life may be selective rather than uniform across cardiometabolic markers. In particular, vitamin D deficiency appeared to be more closely related to markers of long-term glycemic control, triglyceride metabolism, and central adiposity than to other metabolic domains.

One notable finding was that vitamin D deficiency remained associated with HbA1c, but not with fasting glucose, in the fully adjusted model. This distinction suggests that vitamin D status may be more closely related to long-term glycemic regulation than to short-term glucose fluctuations. This interpretation is supported by recent intervention evidence showing that vitamin D supplementation can improve glycemic control in type 2 diabetes, including HbA1c-related outcomes, although the magnitude of benefit varies across studies [18]. In addition, an updated 2024 systematic review and meta-analysis in older adults reported that low 25(OH)D was associated with incident diabetes even after adjustment for relevant confounders, supporting the view that low vitamin D status is linked to poorer long-term glycemic health in aging populations [19]. From a pathophysiological perspective, vitamin D may influence chronic glycemic status through its role in pancreatic β-cell function, calcium-mediated insulin secretion, and systemic insulin sensitivity [20]. In contrast, fasting glucose is a single-time-point measure and may be more sensitive to recent metabolic conditions, fasting-related variability, and circadian timing than HbA1c [21]. HbA1c, by contrast, reflects average glycemic exposure over approximately 8–12 weeks [22]. Therefore, the discrepancy observed in the present study may reflect differences in the temporal characteristics of glycemic markers rather than inconsistency in biological effects. Taken together, the present HbA1c finding appears consistent with prior evidence. In contrast, the null finding for fasting glucose may indicate that vitamin D status is more closely related to cumulative glycemic exposure than to a single fasting measurement. Given the clinical importance of glycemic control in older adults, the observed association with HbA1c may be clinically meaningful [23].

The association with triglycerides is also consistent with previous findings. A 2024 systematic review and meta-analysis of randomized controlled trials in people with type 2 diabetes concluded that vitamin D supplementation improved triglyceride and HDL levels, while not significantly improving LDL or total cholesterol [24]. Likewise, a 2025 meta-analysis in overweight or obese women reported a significant effect of vitamin D supplementation on hypertriglyceridemia, but not on LDL-C [25]. In the present study, however, only triglycerides remained significant after full adjustment, whereas HDL-cholesterol did not. This divergence suggests that vitamin D deficiency may be preferentially linked to triglyceride metabolism rather than to all lipid fractions. Mechanistically, triglycerides are closely associated with insulin resistance and hepatic lipid synthesis, both of which have been proposed as pathways influenced by vitamin D status. In contrast, HDL-cholesterol is affected by a broader range of genetic, hormonal, and lifestyle factors, which may attenuate its independent association with vitamin D after adjustment. Therefore, the present findings support a more targeted interpretation of vitamin D in lipid metabolism, emphasizing its potential role in triglyceride regulation.

Another important finding was that vitamin D deficiency was associated with waist circumference, but not with body mass index, after adjustment. This distinction highlights the need to differentiate between central adiposity and general obesity in older populations. A 2025 cross-sectional study in older adults demonstrated that serum vitamin D levels were inversely associated with visceral adipose tissue mass, a metabolically active fat compartment strongly linked to insulin resistance and cardiometabolic risk [26]. That study further reported attenuation of the association after adjustment, indicating that adiposity-related relationships are influenced by multiple confounding variables [26]. In the present study, the persistence of waist circumference but not BMI suggests that vitamin D deficiency may be more closely related to abdominal fat distribution than to overall body mass. This finding is particularly relevant in older adults, where age-related changes such as sarcopenia and fat redistribution may limit the interpretability of BMI. Waist circumference, as a marker of central adiposity, may therefore provide a more sensitive indicator of metabolically relevant fat accumulation in this population.

By contrast, the loss of significance for fasting glucose, HDL-cholesterol, and C-reactive protein after multivariable adjustment suggests that the crude associations observed in unadjusted analyses were at least partly explained by confounding variables. Factors such as age, physical activity, smoking, and comorbid conditions are known to influence both vitamin D status and metabolic health, and their inclusion in the adjusted models likely reduced spurious associations. This interpretation is consistent with recent literature showing that vitamin D supplementation may influence inflammatory markers in specific populations rather than uniformly across all adults [27]. Therefore, the absence of an independent association with C-reactive protein in the present study should be interpreted cautiously and may reflect the influence of confounding factors rather than the absence of any relationship. The lack of significant associations for several biomarkers after full adjustment further suggests that the crude relationships observed in unadjusted analyses may have been influenced by confounding factors, highlighting the importance of appropriate adjustment in epidemiological studies.

The cross-sectional design of this study requires cautious interpretation of the observed associations. A 2025 bidirectional two-sample Mendelian randomization study found no evidence that circulating 25(OH)D is linked to metabolic syndrome traits in a directional manner; instead, it suggested that vitamin D status and metabolic traits such as waist circumference, BMI, and triglycerides may be interrelated [28]. This finding is relevant to the present study, as it suggests that the observed associations may reflect complex or bidirectional relationships rather than a simple one-way pathway between vitamin D deficiency and metabolic dysfunction. Taken together, these findings suggest that vitamin D deficiency may be linked to specific aspects of metabolic dysregulation—particularly long-term glycemic control, triglyceride metabolism, and central adiposity—while other associations may be partly influenced by confounding variables. This domain-specific pattern may help explain the heterogeneity observed in previous studies and highlights the need for more targeted investigation of vitamin D-related metabolic pathways. In addition, renal function, which plays an important role in vitamin D metabolism, was not included in the present analysis; therefore, residual confounding variables cannot be excluded. Furthermore, seasonal variation in vitamin D levels was not explicitly accounted for in this study. Although KNHANES employs a year-round rolling survey design, differences in sunlight exposure across seasons may have influenced serum vitamin D concentrations, and thus residual confounding variables related to seasonality may remain. Future studies incorporating kidney function indicators and seasonal adjustment are warranted to better clarify these relationships.

Overall, the present findings should be interpreted cautiously. In this nationally representative sample of older adults, vitamin D deficiency was independently associated with HbA1c, triglycerides, and waist circumference, but not with fasting glucose, HDL-cholesterol, C-reactive protein, or body mass index after full adjustment. These findings are consistent with previous evidence suggesting that the associations between vitamin D deficiency and metabolic markers may differ according to the specific outcome examined.

## 5. Conclusions

Vitamin D deficiency was independently associated with higher HbA1c, triglyceride levels, and waist circumference in older adults, but not with fasting glucose, HDL-cholesterol, C-reactive protein, or body mass index after adjustment. These findings suggest that the associations between vitamin D deficiency and metabolic markers in older adults may differ according to the specific biomarker examined. Future longitudinal and interventional studies are needed to clarify the directionality and underlying mechanisms of these associations.

## Figures and Tables

**Table 1:** General characteristics of participants (*n* = 1806).

Variable	Category	*n*	Weighted % or Mean ± SE	95% CI
Sociodemographic				
Age, years	–	1806	72.78 ± 0.18	72.43–73.14
Sex	Male	760	46.0 ± 1.1	43.9–48.1
	Female	1046	54.0 ± 1.1	51.9–56.1
Residence	Urban	1273	73.8 ± 3.6	66.0–80.4
	Rural	533	26.2 ± 3.6	19.6–34.0
Household income	Q1 (lowest)	737	37.2 ± 1.8	33.8–40.8
	Q2	537	31.4 ± 1.5	28.5–34.5
	Q3	348	20.2 ± 1.4	17.5–23.2
	Q4 (highest)	184	11.2 ± 1.0	9.3–13.4
Education level	≤Elementary	803	43.1 ± 1.7	39.8–46.5
	Middle	373	20.5 ± 1.1	18.5–22.7
	High	412	24.0 ± 1.2	21.7–26.5
	≥College	218	12.4 ± 1.2	10.1–15.0
Health behaviors				
Monthly drinking	No	1186	64.7 ± 1.4	61.9–67.5
	Yes	620	35.3 ± 1.4	32.5–38.1
Current smoking	No	1629	91.9 ± 0.7	90.3–93.2
	Yes	177	8.1 ± 0.7	6.8–9.7
Aerobic physical activity	No	1218	67.6 ± 1.7	64.1–70.9
	Yes	588	32.4 ± 1.7	29.1–35.9
Comorbidities				
Hypertension	No	823	46.0 ± 1.3	43.3–48.6
	Yes	983	54.0 ± 1.3	51.3–56.6
Diabetes mellitus	No	1358	75.1 ± 1.1	72.9–77.2
	Yes	448	24.8 ± 1.1	22.8–27.0
Metabolic and clinical variables				
Vitamin D, ng/mL	–	1806	29.88 ± 0.38	29.13–30.64
Fasting glucose, mg/dL	–	1806	107.42 ± 0.57	106.30–108.54
HbA1c, %	–	1806	5.97 ± 0.02	5.92–6.01
ln (Triglycerides)	–	1806	4.68 ± 0.01	4.66–4.71
ln (C-reactive protein)	–	1806	−0.51 ± 0.03	−0.56 to −0.46
HDL-cholesterol, mg/dL	–	1806	56.41 ± 0.41	55.60–57.21
Waist circumference, cm	–	1806	85.69 ± 0.27	85.17–86.21
Body mass index, kg/m^2^	–	1806	24.07 ± 0.09	23.90–24.24

Note. Values are presented as weighted percentages with standard errors (SE) for categorical variables and weighted means with SE for continuous variables, along with 95% confidence intervals (CI), using a complex sample design. Unweighted frequencies are presented as n. Household income quartiles were defined according to the 2024 KNHANES household income quartile criteria based on monthly equivalized household income: Q1, ≤129.64; Q2, 129.65–264.89; Q3, 264.90–418.34; and Q4, >418.34 (10,000 KRW/month). Triglycerides and C-reactive protein were log-transformed due to skewed distributions and are presented as ln-transformed values. Abbreviations: SE, standard error; CI, confidence interval; HbA1c, glycated hemoglobin; HDL, high-density lipoprotein; ln, natural logarithm.

**Table 2:** Comparison of characteristics according to vitamin D status.

Variable	Category	Deficient (<20 ng/mL, *n* = 409)	Non-Deficient (≥20 ng/mL, *n* = 1397)	F	*p*
Sociodemographic factors					
Age, years	–	73.37 ± 0.34	72.61 ± 0.18	4.96	0.026
Sex	Male	63.4 (1.8)	40.9 (1.2)	55.35	<0.001
	Female	36.6 (1.8)	59.1 (1.2)		
Residence	Urban	74.7 (2.0)	73.6 (1.4)	0.10	0.748
	Rural	25.3 (2.0)	26.4 (1.4)		
Household income	Q1	38.4 (2.5)	36.9 (1.7)	0.55	0.643
	Q2	32.4 (2.2)	31.1 (1.5)		
	Q3	17.7 (1.7)	20.9 (1.3)		
	Q4	11.5 (1.3)	11.1 (1.0)		
Education level	≤Elementary	43.0 (2.4)	43.1 (1.6)	0.33	0.788
	Middle	19.0 (1.8)	20.9 (1.3)		
	High	24.3 (2.0)	23.9 (1.4)		
	≥College	13.7 (1.5)	12.0 (1.0)		
Health behaviors					
Monthly drinking	No	61.0 (2.4)	65.8 (1.5)	2.57	0.111
	Yes	39.0 (2.4)	34.2 (1.5)		
Current smoking	No	85.4 (1.7)	93.8 (0.8)	22.77	<0.001
	Yes	14.6 (1.7)	6.2 (0.8)		
Aerobic physical activity	No	73.7 (2.1)	65.9 (1.6)	5.48	0.020
	Yes	26.3 (2.1)	34.1 (1.6)		
Comorbidities					
Hypertension	No	45.7 (2.4)	46.1 (1.6)	0.13	0.872
	Yes	54.3 (2.4)	53.9 (1.6)		
Diabetes mellitus	No	72.1 (2.1)	76.1 (1.3)	3.27	0.040
	Yes	27.9 (2.1)	23.9 (1.3)		
Metabolic and clinical variables					
Waist circumference, cm	–	87.26 ± 0.64	85.23 ± 0.31	11.65	<0.001
Body mass index, kg/m^2^	–	24.27 ± 0.15	24.01 ± 0.10	2.21	0.139
HDL-cholesterol, mg/dL	–	52.01 ± 0.77	57.69 ± 0.48	39.58	<0.001
Fasting glucose, mg/dL	–	110.68 ± 1.46	106.47 ± 0.61	6.70	0.010
HbA1c, %	–	6.08 ± 0.05	5.93 ± 0.02	8.50	0.004
ln (Triglycerides)	–	4.79 ± 0.03	4.66 ± 0.01	15.00	<0.001
ln (C-reactive protein)	–	−0.31 ± 0.06	−0.57 ± 0.03	16.00	<0.001

Note. Values are presented as weighted percentages (SE) for categorical variables and weighted means (SE) for continuous variables. The Rao–Scott chi-square test (reported as adjusted F statistics) was used for categorical variables, and complex samples general linear models were used for continuous variables. Household income quartiles were defined according to the 2024 KNHANES household income quartile criteria based on monthly equivalized household income: Q1, ≤129.64; Q2, 129.65–264.89; Q3, 264.90–418.34; and Q4, >418.34 (10,000 KRW/month). Triglycerides and C-reactive protein were log-transformed due to skewed distributions and are presented as ln-transformed values. Abbreviations: HbA1c, glycated hemoglobin; HDL, high-density lipoprotein; ln, natural logarithm.

**Table 3:** Association between vitamin D deficiency and metabolic biomarkers in older adults.

Outcome	Model 1 β (95% CI)	*p*	Model 2 β (95% CI)	*p*	Model 3 β (95% CI)	*p*
Fasting glucose (mg/dL)	4.21 (1.04, 7.39)	0.010	2.87 (−0.34, 6.07)	0.079	2.17 (−1.01, 5.35)	0.180
HbA1c (%)	0.15 (0.05, 0.25)	0.004	0.14 (0.04, 0.24)	0.008	0.10 (0.01, 0.20)	0.034
ln (Triglycerides)	0.13 (0.07, 0.19)	<0.001	0.12 (0.06, 0.19)	<0.001	0.10 (0.04, 0.17)	0.003
ln (C-reactive protein)	0.06 (−0.03, 0.15)	0.196	0.05 (−0.04, 0.14)	0.276	0.04 (−0.05, 0.13)	0.391
HDL-cholesterol (mg/dL)	−1.87 (−3.22, −0.52)	0.007	−1.21 (−2.58, 0.16)	0.083	−0.89 (−2.21, 0.43)	0.186
Waist circumference (cm)	2.03 (0.85, 3.20)	<0.001	1.54 (0.36, 2.72)	0.011	1.21 (0.06, 2.37)	0.040
Body mass index (kg/m^2^)	0.25 (−0.08, 0.59)	0.139	0.19 (−0.15, 0.52)	0.273	0.12 (−0.21, 0.45)	0.471

Note. Model 1 was unadjusted. Model 2 was adjusted for age, sex, residence, household income, and education level. Model 3 was additionally adjusted for smoking, alcohol consumption, aerobic physical activity, hypertension, and diabetes mellitus. β coefficients represent differences in metabolic biomarker levels for participants with vitamin D deficiency (<20 ng/mL) compared with those without deficiency (≥20 ng/mL). Triglycerides and C-reactive protein were log-transformed due to skewed distributions and are presented as ln-transformed values. All analyses were conducted using complex sample design procedures. Abbreviations: CI, confidence interval; HbA1c, glycated hemoglobin; HDL, high-density lipoprotein; ln, natural logarithm.

## Data Availability

The data used in this study are publicly available from the Korea National Health and Nutrition Examination Survey (KNHANES) and can be accessed at https://knhanes.kdca.go.kr (accessed on 28 March 2026) upon reasonable request and approval by the Korea Disease Control and Prevention Agency.

## Abbreviations

The following abbreviations are used in this manuscript:

25(OH)D	25-hydroxyvitamin D
BMI	body mass index
CI	confidence interval
CRP	C-reactive protein
HbA1c	glycated hemoglobin
HDL-C	high-density lipoprotein cholesterol
IBM	International Business Machines
IRB	Institutional Review Board
KDCA	Korea Disease Control and Prevention Agency
KNHANES	Korea National Health and Nutrition Examination Survey
SE	standard error
SPSS	Statistical Package for the Social Sciences
